# Beyond linear: How circRNAs twist and turn Notch signaling

**DOI:** 10.1002/ccs3.70038

**Published:** 2025-08-03

**Authors:** Pegah Yazdan Panah, Amir Sadeghi, Soudeh Ghafouri‐Fard

**Affiliations:** ^1^ Student Research Committee School of Medicine Shahid Beheshti University of Medical Sciences Tehran Iran; ^2^ Gastroenterology and Liver Diseases Research Center Research Institute for Gastroenterology and Liver Diseases Shahid Beheshti University of Medical Sciences Tehran Iran; ^3^ Department of Medical Genetics Shahid Beheshti University of Medical Sciences Tehran Iran

**Keywords:** cancer, circRNA, lncRNA, non‐coding RNA, Notch pathway

## Abstract

Circular RNAs (circRNAs) have emerged as pivotal regulators of the Notch signaling pathway, influencing diverse pathological processes ranging from cancer to neurodegenerative disorders. This review synthesizes evidence demonstrating how circRNAs modulate Notch activity through miRNA sponging (e.g., circ‐NOTCH 1 promoting gastric cancer metastasis via miR‐637/apelin axis), protein interactions, and peptide encoding. Key examples of oncogenic circRNAs are circNFIX (glioma) and circ‐ASH2L (pancreatic cancer), which drive tumor progression by sponging miR‐34a‐5p, elevating NOTCH 1 expression, and activating downstream effectors. We also discuss tissue‐specific duality of circRNAs. In fact, Notch signaling exhibits context‐dependent roles, with circFBXW7 suppressing NOTCH 1 in T‐ALL (tumor suppressor) versus circ‐NSD2 activating JAG1/NOTCH 1 in colorectal cancer (oncogene). While circRNAs like hsa_circ_0001741 show prognostic promise, challenges persist in delivery and target specificity due to miRNA pleiotropy. By integrating mechanistic insights with preclinical examples, this review highlights circRNAs as both biomarkers and therapeutic targets, urging further research to address clinical translation barriers.

## Graphical abstract

Circular RNAs have crucial functions in the regulation of the Notch pathway. They mainly regulate this pathway through modulation of NOTCH 1, NOTCH 2, and NOTCH 3. Jagged and DLL transcripts are also regulated by circular RNAs.

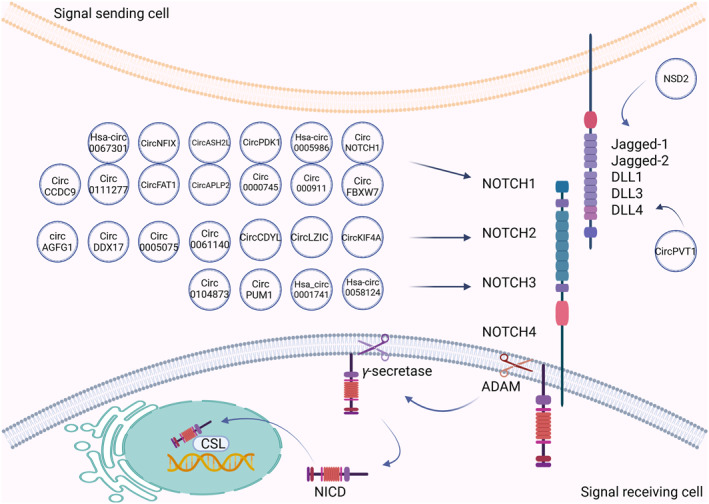

## 1 INTRODUCTION

**FIGURE 1:**
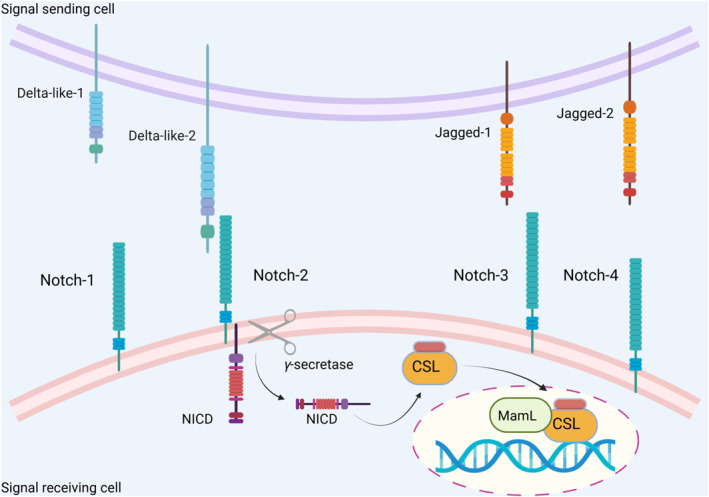
The key steps of the activation of Notch receptors and how they trigger downstream signaling events.

As an evolutionarily conserved intercellular communication system, the Notch signaling pathway serves crucial roles in cell differentiation, proliferation, apoptosis, and stem cell maintenance.^1^ This pathway is regarded as a pillar of juxtacrine signaling that arranges complex intercellular communication, controlling different developmental and homeostatic processes through a firmly orchestrated cascade of enzymatic cleavage reactions.^1^ This pathway results in the nuclear transposition of the Notch intracellular domain (NICD) and the successive induction of downstream target genes. Four Notch receptors (Notch 1, Notch 2, Notch 3, and Notch 4)^2^ and five ligands for these receptors (Dll‐1, Dll‐3, Dll‐4, Jagged 1, and Jagged 2)^3^ have been identified in humans. The interaction between Notch receptors and their ligands results in the activation of the Notch pathway. In fact, this interaction triggers proteolytic cleavage by γ‐secretase, releasing the NICD, which enters the nuclear compartment.^1^ Subsequently, the NICD cooperates with CSL (CBF1/RBP‐Jκ, Su(H), Lag‐1) transcription factors, recruiting coactivators like Mastermind‐like (MAML) to activate expression of a number of targets, including Hes and Hey family members.^1^ Figure 1 illustrates the key steps of how Notch receptors are activated and how they trigger downstream signaling events.

These genes have a crucial impact on a variety of molecular processes, such as cell cycle transition, cellular differentiation, intercellular adhesion, and commitment to specialized cell fates.^4^

Aberrant function of Notch signaling is associated with various disorders, including cancer,^5^ cardiovascular disorders,^6^ neurodegenerative conditions^7^, and reproductive disorders.^4^

In recent years, circular RNAs (circRNAs) have been acknowledged as key regulators of gene expression.^8^ These single‐stranded, covalently closed RNA molecules are extremely stable and display a tissue‐specific expression pattern.^9^ Growing evidence proposes that circRNAs have a significant role in modulating the Notch pathway mainly by acting as microRNA (miRNA) sponges.^10^ Moreover, other mechanisms such as their interaction with RNA‐binding proteins (RBPs),^11^ or even production of functional peptides might contribute to the regulation of certain signaling pathways.^12^

This article explores the mechanisms through which circRNAs regulate the Notch pathway and their implications in disease and therapy.

## 2 CircRNAs INTERACTION WITH NOTCH SIGNALING IN CANCERS

### 2.1 CircRNAs interacting with NOTCH 1

Circ‐NOTCH 1 is an upregulated circRNA in gastric cancer. Notably, circ‐NOTCH 1 has multiple binding sites for miR‐637. Additionally, this miRNA targets apelin, another gene that is highly expressed in gastric cancer cells and tissues. Circ‐NOTCH 1 increases cell proliferation and invasiveness, and decreases cell apoptosis through inhibiting the transcriptional activity of miR‐637, thus increasing apelin levels.^13^ Similarly, circ‐NOTCH 1 antagonizes miR‐449c‐5p availability to facilitate MYC‐induced NOTCH 1 overexpression that eventually leads to induction of metastasis and stem cell properties in gastric cancer.^14^ Besides, c‐Myc‐induced circ‐NOTCH 1 enhances aggressiveness of nasopharyngeal carcinoma cells through modulating the miR‐34c‐5p/c‐Myc axis.^15^

circNFIX is another circRNA that is overexpressed in glioma tissues compared with corresponding normal tissue samples consistent with upregulation of the Notch signaling pathway. Notably, circNFIX acts as a sponge for miR‐34a‐5p, an miRNA that targets NOTCH 1. Both silencing of circNFIX and overexpression of miR‐34a‐5p inhibit cell propagation and migration. Besides, an miR‐34a‐5p inhibitor could neutralize the inhibitory effect of siRNA designed against circNFIX (si‐circNFIX) on glioma cells. As in vivo assays show, si‐circNFIX inhibits glioma growth through regulation of miR‐34a‐5p and NOTCH 1.^16^

Expression of NOTCH 1 and hsa_circ_0005986 as a NOTCH 1 regulatory circRNA has been affected by an extremely low‐frequency magnetic field (ELF‐MFs). Notably, MFs could decrease the viability of gastric tumor cells, while increasing the viability human normal fibroblast. Besides, all magnetic flux densities (MFDs) have downregulated NOTCH 1 in gastric tumor cells and upregulated its expression in normal fibroblasts, dependent on the MFD of MFs. In fact, the tumor and normal cells have exhibited distinct molecular behavior in different MFDs regarding NOTCH 1 and hsa_circ_0005986 levels. Reduction of tumor cells survival after exposure to ELF‐MFs might be due to downregulation of NOTCH 1 and hsa_circ_0005986.^17^

A high throughput circRNA analysis has resulted in the identification of circRNA‐000911 as a downregulated circRNA in breast cancer cells.^18^ Functional studies have shown that overexpression of circRNA‐000911 inhibits proliferation, migration, and invasion, and promotes apoptosis.^18^ RNA precipitation assay has suggested miR‐449a as the circRNA‐000911‐associated miRNA. Mechanistically, miR‐449a antagonizes circRNA‐000911 to affect progression of breast cancer.^18^ Most notably, Notch 1 is a target of miR‐449a, through which circRNA‐000911 influences breast cancer progression. Besides, NF‐κB signaling is functionally influenced by the circRNA‐000911/miR‐449a axis.^18^ Taken together, circRNA‐000911 has an anti‐oncogenic effect in breast cancer and might be used as a therapeutic target in this cancer.^18^

Originated from exons 1 and 2 of the *NSD2* gene, circ‐NSD2 is overexpressed in colorectal cancer tissues, particularly in advanced stages or metastatic tumors.^19^ This circRNA increases the migration, invasiveness, and metastasis of cancer cells through targeting miR‐199b‐5p and subsequent regulation of DDR1 and Jagged 1.^19^ The latter is regarded as a ligand for NOTCH 1 and is implicated in epithelial mesenchymal transition in colorectal cancer.^20^ In fact, DDR1 and Jagged 1 have a synergic effect on regulation of cell–matrix interactions, migration, and metastasis of colorectal cancer.^19^ Table 1 shows circRNAs that affect NOTCH 1 expression in different cancers.

**TABLE 1:** Role of circRNAs in the regulation of NOTCH 1 expression in cancers.

Circular RNA	Regulation	Mechanism	Effect on Notch pathway	Biological outcome	Disease	References
CircNFIX	Upregulation	Sponges miR‐34a‐5p	↑ Notch 1 expression	Oncogenic function	Glioma	16
CircFAT1	Upregulation	Sponges miR‐525‐5p	↑ Notch 1 expression	Promotes cancer cell metastasis and chemoresistance	Breast cancer	21
Circ‐ASH2L	Upregulation	Sponges miR‐34a	↑ Notch 1 expression	Promotes tumor invasion, proliferation and angiogenesis	Pancreatic ductal adenocarcinoma	22
CircAPLP2	Upregulation	Sponges miR‐101‐3p	↑ Notch 1 expression	Promotes proliferation and metastasis	Colorectal cancer	10
CircPVT1	Upregulation	Sponges miR‐30e	↑ DLL4/Notch expression	Promotes cell proliferation and inhibits cell apoptosis	T cell acute lymphoblastic leukemia (T‐ALL)	23
CircPDK1	Upregulation	Sponges miR‐377‐3P	↑ Notch 1 expression	Promotes cell migration and invasion	Renal cell carcinoma	24
Circ_0000745	Upregulation	Sponges miR‐193b‐3p	↑ Notch 1 expression	Promotes cell proliferation and inhibits cell apoptosis	Pediatric T cell acute lymphoblastic leukemia	25
Circ‐NOTCH 1	Upregulation	Sponges miR‐449c‐5p	↑ Notch 1 expression	Promotes metastasis and stemness	Gastric cancer	14
Circ‐UBR5	Upregulated	Sponges miR‐1179 and upregulates UBR5	↑ Notch 1 and 4 expression	Induces growth and metastasis	Triple‐negative breast cancer	26, 27
Circ‐NSD	Upregulation	Sponges miR‐199b‐5p	↑ DDR1/JAG1 expression	Promotes invasion and metastasis	Colorectal cancer	19
Circ_0008532	Upregulation	Sponge for miR‐155‐5p/miR‐330‐5p	↑ MTGR1	Improves cell migration, invasion, and angiogenesis	Bladder cancer	28
			↓ Notch 1			
Circ_0084582	Upregulation	Sponges miR‐485‐3p	↑ JAG1	Increases cell growth, migration, invasion, and angiopoiesis	Osteosarcoma	29
Hsa_circ_0005986	Downregulation	Sponges miR‐129‐5p	↑ Notch 1 expression	Affects proliferation through regulating the G0/G1 to S transition	Hepatocellular carcinoma	30
Circ‐000911	Downregulation	Sponges miR‐449a	↓ Notch 1 expression	Suppresses cell proliferation	Breast cancer	18
CircFBXW7	—	—	↓ Notch 1 expression	Decreases cell viability and reduces MYC activity	T‐ALL	31

### 2.2 CircRNAs interacting with NOTCH 2

circKIF4A (hsa_circ_0007255) is an example of circRNAs contributing to the development and progression of bladder cancer through modulating NOTCH 2 expression. Its expression has been significantly enhanced in clinical samples and cell lines originated from this type of cancer. Notably, its knockdown has led to inhibition of the proliferation and colony formation capacity of bladder cancer cells. In addition, migration and metastatic aptitude of cancer cells have been dramatically reduced after circKIF4A silencing. This circRNA sponges miR‐375/1231 to stimulate bladder cancer progression through enhancing expression of NOTCH 2.^32^ Table 2 shows circRNAs that affect NOTCH 2 expression in different cancers.

**TABLE 2:** Role of circRNAs in the regulation of NOTCH 2 expression in cancers.

Circular RNA	Regulation	Mechanism	Effect on Notch pathway	Biological outcome	Disease	References
CircKIF4A (hsa_circ_0007255)	Upregulation	Sponges miR‐375/1231	↑ Notch 2 expression	Enhances tumor proliferation, colony formation migration, and metastasis	Bladder cancer	32
CircCDYL (hsa_circ_20406)	Upregulation	Sponges miR‐892a and miR‐328‐3p	↑HIF1AN/Notch 2	Plays a role in defining the characteristics of EpCAM‐positive liver cancer stem cells	Hepatocellular carcinoma	33

### 2.3 CircRNAs interacting with NOTCH 3

Circ_0000043 (circ_PUM1) is an example of circRNAs contributing to the progression of endometrial cancer through regulation of NOTCH 3 expression. This circRNA has been expressed at expressively higher level in endometrial cancer samples than in normal samples. Its upregulation promotes proliferation, migration, and invasion of endometrial carcinoma cells. Mechanistically, circ_PUM1 binds to miR‐136, thus increasing its target gene NOTCH 3. These effects can be inverted by upregulation of miR‐136, suggesting the functional role of circ_PUM1/miR‐136/NOTCH3 axis in the development of endometrial cancer.^34^ CircRNA‐mediated modulation of NOTCH 3 is also involved in the pathogenesis of esophageal squamous cell carcinoma (ESCC). Being upregulated in ESCC tissues, hsa_circ_0001741 is positively associated with lymphatic metastasis, high TNM stage, and poor clinical outcome. Functionally, hsa_circ_0001741 stimulates cancer stemness, invasion, and migration through sequestering the tumor suppressor miRNA miR‐491‐5p. Precisely, hsa_circ_0001741 binds to miR‐491‐5p to preclude its binding to the 3′‐UTR of NOTCH 3 transcript and inhibiting NOTCH 3 expression. Furthermore, hsa_circ_0001741 silencing significantly inhibits the in vivo tumorigenic properties of ESCC cells. Taken together, hsa_circ_0001741 is a potential prognostic marker for ESCC.^35^ Table 3 shows circRNAs that affect NOTCH 3 expression in different cancers.

**TABLE 3:** Role of circRNAs in the regulation of NOTCH 3 expression in cancers.

Circular RNA	Regulation	Mechanism	Effect on Notch pathway	Biological outcome	Disease	References
Circ_PUM1 (circ_0000043)	Upregulation	Sponges miR‐136	↑ Notch 3 expression	Promoted the proliferation, migration, and invasion	Endometrial carcinoma	34
Hsa_circ_0001741	Upregulation	Sponges miR‐491‐5p	↑ Notch 3 expression	Promotes stemness, invasion, and migration	Esophageal squamous cell carcinoma	35
Hsa_circ_0058124	Upregulation	Sponges miR‐218‐5p, thereby relieving NUMB	↓ Notch 3/GATAD2A	Promotes proliferation, tumorigenicity, and invasiveness	Papillary thyroid cancer	36

### 2.4 CircRNAs interaction with Notch signaling in nonmalignant disorders

CircRNA‐mediated regulation of Notch signaling is also involved in the pathogenesis of a variety of nonmalignant disorders (Table 4), such as preeclampsia (PE),^42^ neuropathic pain^43^, endometriosis,^41^ Alzheimer's disease (AD),^49^ viral myocarditis,^45^ and osteoarthritis (OA).^37^ For instance, being upregulated in PE placentas compared to normal pregnancy placenta, hsa_circ_0111277 level is positively correlated with PE‐related parameters. This circRNA is mainly localized within the cytoplasm of trophoblasts where it sponges hsa‐miR‐494‐3p to weaken the repressive effect of this miRNA on HTRA1/Notch 1 levels. In fact, migration and invasion of trophoblasts are regulated by the hsa_circ_0111277/miR‐494‐3p/HTRA1/Notch 1 axis, potentiating this axis as a new therapeutic target for PE.^42^ Additionally, function of circ_0005075 in neuropathic pain has been evaluated in the mice model of chronic constriction injury (CCI) mimicking neuropathic pain in human subjects.^43^ Being appreciated as a key oncogene in several malignancies,^52^, ^53^ it is also regarded as an inflammation‐associated circRNA. Expression of circ_0005075 has been evidently elevated in CCI rat models. Silencing of circ_0005075 has suppressed mechanical and thermal hypersensitivity to pain. Furthermore, circ_0005075 knockdown has repressed the neuroinflammation through targeting COX‐2, IL‐6, and TNF‐α, increasing IL‐10 levels and modulating NOTCH 2. Additional experiments have shown that miR‐151a‐3p is a target of circ_0005075 that is decreased in CCI rats. Taken together, loss of circ_0005075 has reduced neuropathic pain through induction of miR‐151a‐3p and suppression of NOTCH 2.^43^

**TABLE 4:** Involvement of circRNAs in the modulation of the Notch pathway in malignant disorders.

CircularRNA	Regulation	Mechanism	Effect on Notch pathway	Biological outcome	Disease	References
Circ_0104873	Upregulation	Sponges miR‐875‐5p	↑ Notch 3 expression	Contributes to the proliferation and osteogenic differentiation of BMSCs cells	Osteoarthritis	37
CircLZIC	Upregulation	Sponges miR‐330‐5p	↑ Notch 2 expression	Promotes cell proliferation and inhibits apoptosis	Atherosclerosis	38
Circ_0006476	—	Sponges miR‐3074‐5p	Regulates DLL4 and Notch pathway	Regulates macrophage apoptosis	Atherosclerosis	39
Circ_0061140	Upregulation	Binds to miR‐140‐3p	↑ Notch 2 expression	Regulates proliferation, migration, and invasion	Endometriosis	40
Hsa_circ_0067301	Downregulation	Binds to miR‐141–5p	↓ Notch 1 expression	Regulates proliferation, migration, and EMT	Endometriosis	41
Circ_0111277	Upregulation	Sponges hsa‐miR‐494‐3p	↑ HTRA1/Notch 1	Retards trophoblast cells migration and invasion	Preeclampsia	42
Circ_0005075	Upregulation	Sponges miR‐151a‐3p	↑ Notch 2 expression	Contributes to neuropathic pain behaviors and neuroinflammation	Neuropathic pain	43
CircCCDC9	Upregulation	—	↓ Notch 1 expression	Attenuates apoptosis	Cerebral I/R injury	44
CircDDX17	Downregulation	Sponges miR‐1248	↑ Notch 2 expression	Enhances coxsackievirus B3 replication	Viral myocarditis	45
CircHipk3	Upregulation	Inhibits miR‐133a activity	↑ Notch 1 expression	Induces cardiac regeneration and angiogenesis, reduces the infarct size after MI, and may restore cardiac function	Myocardial infarction	46
CircAGFG1	Upregulation	Sponges miR‐1257	Notch 2	Enhances autophagy and reduces apoptosis	Tuberculosis	47
Hsa_circ_0006766	Upregulation	Downregulates hsa‐miR‐4739	↑ Notch 2 expression	Promotes BMSC osteogenic differentiation	—	48
Circ_0004381	Downregulated	Interacts with miR‐647	↓ PSEN1	Promotes survival of hippocampal neurons and polarization of microglia toward the M2 anti‐inflammatory state	Alzheimer's disease	49
CircPSEN1s (hsa_circ_0008521 and chr14:73614502–73614802)	—	Sponge 8 miRNAs, including has‐mir‐4668‐5p and has‐mir‐5584‐5p	—	Interact with FOXA1, ESR1, HNF1B, BRD4, GATA4, EP300, CBX3, PRDM9, and PPARG proteins, which can target the TGF‐β and Notch pathways	Alzheimer's disease	50
CircSOD2	—	Sponges miR‐206	↑ Notch 3 expression	Regulates smooth muscle cell proliferation and neointima formation	Vascular injury	51

CircRNA‐mediated regulation of the Notch pathway is also implicated in the pathogenesis of endometriosis. Notably, expression of hsa_circ_0067301 and miR‐141–5p has been found to be decreased in ectopic endometrium compared to control endometrial samples. Knockdown of hsa_circ_0067301 has led to enhancement of proliferation and migration of Ishikawa and End1/E6E7 cells, along with elevation of expression of Notch 1, Hes‐1, N‐cadherin, and vimentin but reduction of E‐cadherin levels. In fact, hsa_circ_0067301/miR‐141–5p/Notch 1 axis has been shown to exert a crucial regulatory role in the process of epithelial–mesenchymal transition in endometriosis.^41^

While the abovementioned circRNAs have a relatively direct effect on Notch signaling, circ_0004381 affects expression of presenilin 1 (PSEN1),^49^ a key protein involved in the γ‐secretase complex, which is essential for the cleavage of Notch receptors, a process crucial for Notch signaling.^54^ Considering the importance of PSEN1 in the pathogenesis of AD through regulation of the Notch pathway,^55^ it is not surprising that circ_0004381 has a key role in the pathogenesis of AD. In fact, expression of this circRNA has been found to be elevated in Aβ1‐42‐treated hippocampal neurons. Circ_0004381 silencing has alleviated Aβ1‐42‐triggered apoptosis, oxidative damage, and mitochondrial impairment in hippocampal neurons. Moreover, its knockdown has enhanced microglial M2‐type polarization, suppressed the release of inflammatory molecules by microglia, and improved cognitive functions in animal models of AD. From a mechanistical point of view; circ_0004381 regulates PSEN1 expression through sequestering miR‐647, suggesting the importance of the circ_0004381/miR‐647/PSEN1 axis in the pathogenesis of AD through modulation of the Notch pathway.^49^

Additionally, hsa_circ_0063331 (circDDX17) is involved in the pathoetiology of coxsackievirus B3‐induced myocarditis through regulation of NOTCH 2. Expression of this circRNA has been considerably reduced after infection with this virus. Moreover, circDDX17 enhances replication of viral particles through decreasing the expression of miR‐1248, an miRNA that interacts with NOTCH 2. In this context, NOTCH 2 upregulates expression of methyltransferase‐like protein 3, a protein that regulates viral replication.^45^

Finally, circ_0104873 has been identified as an upregulated circRNA during bone marrow mesenchymal stem cells (BMSCs) differentiation toward osteogenic lineage. Mechanistically, circ_0104873 sponges miR‐875‐5p to increase expression of NOTCH 3, thus stimulating the Notch signaling pathway. Notably, this circRNA contributes to the development of OA through modulating the miR‐875‐5p/NOTCH 3/Notch axis.^37^

## 3 DISCUSSION

Personalized medicine has been emerged as a novel modality to precise and targeted therapy of cancer.^56^ This field involves modulation of certain signaling pathways as well as the noncoding region of the genome.^57^ A practical strategy to inhibit the development and spread of tumors is to interfere with the Notch signaling pathways. Novel approaches to specifically hinder pro‐tumorigenic functions of this pathway can be developed through modulation of the function of circRNAs. Thus, circRNAs that regulate the Notch pathway have therapeutic potential in cancer. In the vast majority of cases, overexpression of oncogenic circRNAs enhances Notch signaling, promoting tumor growth. Conversely, tumor‐suppressive circRNAs inhibit Notch‐driven malignancies. However, there are some exceptions to this rule. For instance, the oncogenic circRNA, circ_0008532 enhances bladder cancer cells migration, invasion, and angiogenesis through inhibiting the Notch signaling pathway.^28^ It is worth mentioning that the Notch pathway can exert both oncogenic and tumor suppressor functions depending on cell context as well as the genetic landscape inside a particular cell type.^58^ In fact, the Notch pathway's functional duality is orchestrated by circRNAs in a tissue‐ and disease‐specific manner. CircRNA stoichiometry and competing endogenous RNA (ceRNA) networks may determine Notch's role, with high circRNA levels favoring oncogenic outcomes in proliferative tissues.

A recurring theme across both cancers and nonmalignant diseases is the sequestration of tumor‐suppressive miRNAs by circRNAs, leading to derepression of Notch signaling. Key examples include miR‐34 and miR‐449 families. CircNFIX and circ‐ASH2L sponge miR‐34a‐5p, elevating NOTCH 1 expression and driving proliferation of glioma^16^ and pancreatic cancer cells,^22^ respectively. Circ‐NOTCH 1 (gastric cancer)^14^ and circ‐000911 (breast cancer)^18^ antagonize miR‐449c‐5p and miR‐449a, respectively, amplifying NOTCH 1 activity and metastasis. These conserved miRNA–circRNA interactions suggest that therapeutic targeting of specific miRNA nodes (e.g., miR‐34 or miR‐449) could simultaneously disrupt Notch signaling in diverse pathologies.

**FIGURE 2:**
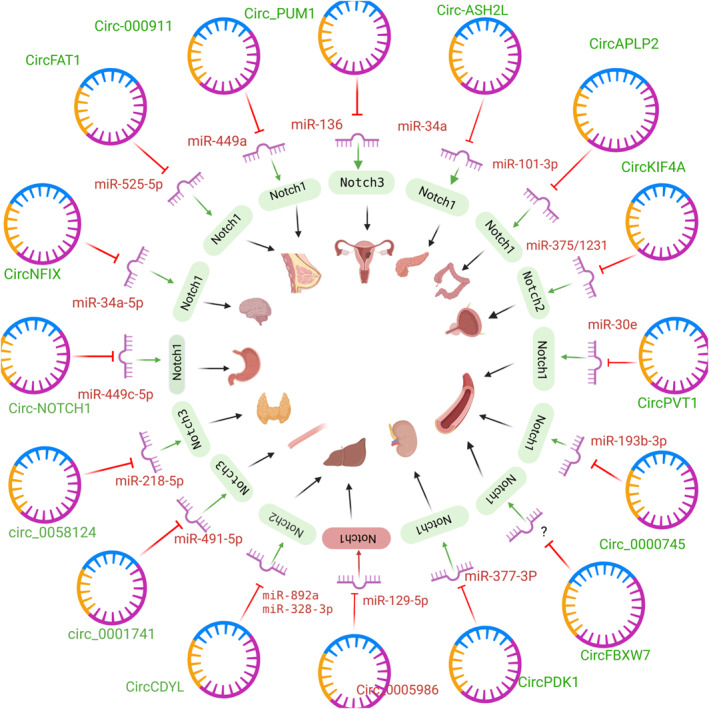
Overview of circRNAs that regulate different Notch receptors through miRNA sponging.

While the role of NOTCH 1‐interacting circRNAs has been more assessed in the pathogenesis of cancers, circRNAs that interact with other NOTCH receptors or ligands have been less studied. Figure 2 shows an overview of circRNAs that regulate different Notch receptors.

Despite tissue‐specific contexts, circRNA‐mediated Notch activation converges on common downstream effectors. For instance, DLL/JAG ligands are examples of these downstream effectors. Circ‐NSD2 upregulates JAG1 in colorectal cancer,^19^ while circ_0006476 modulates DLL4 in atherosclerosis,^39^ illustrating how circRNAs regulate Notch ligands to amplify pathway activity across diseases. Targeting these downstream effectors (e.g., with γ‐secretase inhibitors) may circumvent tissue‐specific circRNA heterogeneity.

CircRNAs are emerging as important regulators of the Notch pathway through miRNA sponging, interaction with some proteins, and perhaps in rare cases, peptide encoding. However, the latter function has not been well‐established in this context. Their dysregulation contributes to various diseases, particularly cancers; thus, they are attractive targets for therapy.

Current research on NOTCH‐interacting circRNAs has predominantly focused on their oncogenic or tumor‐suppressive roles in cancer. However, their implications in nonmalignant conditions remain underexplored. To date, only a handful of disorders have been investigated in this context, including PE,^42^ neuropathic pain^43^, endometriosis,^41^ AD,^49^ viral myocarditis,^45^ and OA.^37^ The narrow scope of studied diseases highlights a significant knowledge gap, particularly in cardiovascular, metabolic, and autoimmune disorders. Besides, most studies have correlated circRNA expression changes with disease states in animal models or cell lines but lack validation in human samples. Whether targeting NOTCH‐interacting circRNAs could ameliorate these conditions remains unexplored. In fact, expanding research into nonmalignant diseases could uncover novel circRNA‐dependent processes and therapeutic prospects associated with the NOTCH pathway.

It is worth mentioning that circRNAs exploit Notch signaling to drive hallmark cancer phenotypes, which overlap with nonmalignant disorders. EMT and metastasis are important examples in this regard. Circ‐NSD2 (colorectal cancer)^19^ and hsa_circ_0067301 (endometriosis)^41^ both regulate Notch to modulate expressions of E‐cadherin and N‐cadherin/vimentin.

Stemness is another feature shared between these two conditions that might be subject of circRNA‐mediated regulation of the Notch pathway. Besides, angiogenesis as another important shared feature between malignant and nonmalignant conditions has been shown to be affected by circ_0084582 and circHipk3 in the contexts of osteosarcoma^29^ and myocardial infarction,^46^ through modulation of VEGF and DLL4, respectively. These shared hallmarks suggest that circRNA–Notch interactions are not disease‐specific but rather represent universal regulatory modules adaptable to different pathological contexts.

The summarized data in this article show that synthetic circRNAs or antisense oligonucleotides can be designed to modulate Notch signaling. For instance, silencing circNFIX in glioma models has dramatically reduced tumor growth via miR‐34a‐5p/NOTCH 1 inhibition.^16^ Besides, overexpression of circ_000911 has suppressed breast cancer xenografts by restoring the miR‐449a/Notch 1 axis.^18^

Small molecules or antisense oligonucleotides against recurrent miRNA sponges (e.g., miR‐34a‐5p) could disrupt multiple circRNA–Notch axes. Moreover, combination of γ‐secretase inhibitors with circRNA‐targeting therapies may enhance efficacy. However, challenges remain in delivering circRNA modulators selectively to tumor or diseased tissues without off‐target effects. Since naked circRNAs are degraded rapidly, exosome‐encapsulated circRNAs or lipid nanoparticles can be used for their delivery. A possible source for off‐target effects is miRNA pleiotropy. Application of tissue‐specific promoters or CRISPR‐based circRNA editing might be regarded as possible solutions for this obstacle. Future research should focus on translating these findings into clinical applications, offering new avenues for personalized medicine.

Besides, the discovery of circRNAs linked to tumors caused by dysregulation of the Notch pathway may provide imperative means for early diagnosis and prognosis. Research has shown that a number of these circRNAs, such as hsa_circ_0001741 could serve as prognostic markers in cancer.^35^ Additionally, hsa_circ_0005986** has exhibited prognostic value in hepatocellular carcinoma;^30^ however, research is needed to evaluate its abundance in biofluids and introduce ultrasensitive detection methods for its quantification. Similarly, hsa_circ_0001741 levels correlate with advanced stage and poor survival in ESCC, supporting its use as liquid biopsy target.^35^ The prognostic or diagnostic prospects for other circRNAs should be uncovered in future studies.

Finally, it is worth mentioning that virtually all covered interactions described in this review were restricted to the miRNA sponging activity of circRNAs, neglecting other functions of circRNAs (e.g., protein scaffolding, alternative splicing regulation, or peptide translation). Future studies should also consider these less‐characterized yet important activities of circRNAs. Moreover, it is necessary to assess whether certain circRNAs can affect Notch ligand trafficking or post‐translational modifications. Another unexplored area in the field of circRNAs effects on the Notch pathway is the interaction between circRNAs and natural therapeutic compounds. Recently, natural compounds and proteasome targeting drugs have been suggested as possible treatment modalities for cancer.^59^, ^60^, ^61^ Thus, the effects of these compounds on Notch‐modulating circRNAs should be investigated in future studies.

## 4 FUTURE PERSPECTIVES

Development of targeted therapies to interfere with cancer‐promoting function of Notch is a feasible option in the dawn of precision medicine. Interference with circRNAs that regulates this pathway is among treatment alternatives that specifically target indispensable constituents of the Notch signaling system. A more ample understanding of the spatiotemporal evolving patterns of Notch activity within the tumor niche maybe acquired through incorporating advanced sequencing methods such as single‐cell sequencing and functional assays. Considering the NOTCH's druggability (e.g., γ‐secretase inhibitors),^62^ future efforts should focus on translational studies to unravel the clinical relevance of dual manipulation of the Notch pathway with such drugs and circRNA‐targeting strategies.

While the role of circRNAs in Notch regulation is increasingly recognized, challenges remain. More studies are required to clarify specific circRNA–Notch interactions. Moreover, in vivo models are required to confirm therapeutic potential of manipulation of circRNAs. Finally, developing circRNA‐based drugs needs overcoming delivery and stability issues.

## AUTHOR CONTRIBUTIONS

Pegah Yazdanpanah searched the literature, and designed tables and figures. Amir Sadeghi contributed to the study design and supervision. Soudeh Ghafouri‐Fard supervised the study, wrote the manuscript, and revised it.

## CONFLICT OF INTEREST STATEMENT

The authors declare no conflicts of interest.

## ETHICS STATEMENT

Not applicable (no animal or clinical study).

## Data Availability

Not applicable (No data were used).

## References

[1] Zhou, B. , W. Lin , Y. Long , Y. Yang , H. Zhang , K. Wu , and Q. Chu . 2022. “Notch Signaling Pathway: Architecture, Disease, and Therapeutics.” Signal Transduction and Targeted Therapy 7(1): 95. 10.1038/s41392-022-00934-y. 35332121 PMC8948217

[2] Mumm, J. S. , and R. Kopan . 2000. “Notch Signaling: From the Outside in.” Developmental Biology 228(2): 151–165: PubMed PMID: 11112321. Epub 2000/12/09. eng. 10.1006/dbio.2000.9960. 11112321

[3] Lai, E. C. 2004. “Notch Signaling: Control of Cell Communication and Cell Fate.” Development 131(5): 965–973: PubMed PMID: 14973298. Epub 2004/02/20. eng. 10.1242/dev.01074. 14973298

[4] Parambath, S. , N. R. Selvraj , P. Venugopal , and R. Aradhya . 2024. “Notch Signaling: an Emerging Paradigm in the Pathogenesis of Reproductive Disorders and Diverse Pathological Conditions.” International Journal of Molecular Sciences 25(10): 5423: PubMed PMID: 38791461. Pubmed Central PMCID: PMC11121885. Epub 2024/05/25. eng. 10.3390/ijms25105423. 38791461 PMC11121885

[5] Aithal, M. G. S. , and N. Rajeswari . 2013. “Role of Notch Signalling Pathway in Cancer and Its Association with DNA Methylation.” Journal of Genetics 92(3): 667–675: PubMed PMID: 24371188. Epub 2013/12/29. eng. 10.1007/s12041-013-0284-5. 24371188

[6] Luxán, G. , J. C. Casanova , B. Martínez‐Poveda , B. Prados , G. D'Amato , D. MacGrogan , A. Gonzalez‐Rajal , et al. 2013. “Mutations in the NOTCH Pathway Regulator MIB1 Cause Left Ventricular Noncompaction Cardiomyopathy.” Nature Medicine 19(2): 193–201: PubMed PMID: 23314057. Epub 2013/01/15. eng. 10.1038/nm.3046. 23314057

[7] Kapoor, A. , and A. Nation . 2021. “Role of Notch Signaling in Neurovascular Aging and Alzheimer's disease.” Seminars in Cell & Developmental Biology 116: 90–97: PubMed PMID: 33384205. Pubmed Central PMCID: PMC8236496. Epub 2021/01/02. eng. 10.1016/j.semcdb.2020.12.011. 33384205 PMC8236496

[8] Youness, R. A. , H. A. Hassan , T. Abaza , A. A. Hady , H. M. El Magdoub , M. Ali , J. Vogel , et al. 2024. “A Comprehensive Insight and in Silico Analysis of Circrnas in Hepatocellular Carcinoma: a Step Toward Ncrna‐based Precision Medicine.” Cells 13(15): 1245: PubMed PMID: 39120276. Pubmed Central PMCID: PMC11312109. Epub 2024/08/09. eng. 10.3390/cells13151245. 39120276 PMC11312109

[9] Kristensen, L. S. , M. S. Andersen , L. V. W. Stagsted , K. K. Ebbesen , T. B. Hansen , and J. Kjems . 2019. “The Biogenesis, Biology and Characterization of Circular Rnas.” Nature Reviews Genetics 20(11): 675–691: PubMed PMID: 31395983. Epub 2019/08/10. eng. 10.1038/s41576-019-0158-7. 31395983

[10] Wu, H.‐B. , S.‐S. Huang , C.‐G. Lu , S.‐D. Tian , and M. Chen . 2020. “CircAPLP2 Regulates the Proliferation and Metastasis of Colorectal Cancer by Targeting miR‐101‐3p to Activate the Notch Signalling Pathway.” Am J Transl Res 12(6): 2554–2569: PubMed PMID: 32655790. Pubmed Central PMCID: PMC7344090. Epub 2020/07/14. eng. 32655790 PMC7344090

[11] Huang, A. , H. Zheng , Z. Wu , M. Chen , and Y. Huang . 2020. “Circular RNA‐Protein Interactions: Functions, Mechanisms, and Identification.” Theranostics 10(8): 3503–3517: PubMed PMID: 32206104. Pubmed Central PMCID: PMC7069073. Epub 2020/03/25. eng. 10.7150/thno.42174. 32206104 PMC7069073

[12] Meng, E. , J. Deng , R. Jiang , and H. Wu . 2022. “CircRNA‐Encoded Peptides or Proteins as New Players in Digestive System Neoplasms.” Frontiers in Oncology 12: 944159: PubMed PMID: 35936754. Pubmed Central PMCID: PMC9355255. Epub 2022/08/09. eng. 10.3389/fonc.2022.944159. 35936754 PMC9355255

[13] Guan, E. , X. Xu , and F. Xue . 2020. “circ‐NOTCH1 Acts as a Sponge of miR‐637 and Affects the Expression of Its Target Gene Apelin to Regulate Gastric Cancer Cell Growth.” Biochemistry and cell biology = Biochimie et biologie cellulaire 98(2): 164–170: PubMed PMID: 31276627. Epub 2019/07/06. eng. 10.1139/bcb-2019-0079. 31276627

[14] Zhao, X. , Q. Zhong , X. Cheng , S. Wang , R. Wu , X. Leng , and L. Shao . 2020. “miR‐449c‐5p Availability Is Antagonized by circ‐NOTCH1 for MYC‐Induced NOTCH1 Upregulation as Well as Tumor Metastasis and Stemness in Gastric Cancer.” Journal of Cellular Biochemistry 121(10): 4052–4063: PubMed PMID: 31943342. Epub 2020/01/17. eng. 10.1002/jcb.29575. 31943342

[15] Huang, W. , W. Song , Y. Jiang , L. Chen , and H. Lu . 2021. “c‐Myc‐induced circ‐NOTCH1 Promotes Aggressive Phenotypes of Nasopharyngeal Carcinoma Cells by Regulating the miR‐34c‐5p/c‐Myc Axis.” Cell Biology International 45(7): 1436–1447: PubMed PMID: 33675278. Epub 2021/03/07. eng. 10.1002/cbin.11582. 33675278

[16] Xu, H. , Y. Zhang , L. Qi , L. Ding , H. Jiang , and H. Yu . 2018. “NFIX Circular RNA Promotes Glioma Progression by Regulating miR‐34a‐5p via Notch Signaling Pathway.” Frontiers in Molecular Neuroscience 11: 225: PubMed PMID: 30072869. Pubmed Central PMCID: PMC6058096. Epub 2018/08/04. eng. 10.3389/fnmol.2018.00225. 30072869 PMC6058096

[17] Mansoury, F. , N. Babaei , S. Abdi , M. Entezari , and A. Doosti . 2021. “Changes in NOTCH1 Gene and Its Regulatory Circrna, hsa_circ_0005986 Expression Pattern in Human Gastric Adenocarcinoma and Human Normal Fibroblast Cell Line Following the Exposure to Extremely Low Frequency Magnetic Field.” Electromagnetic Biology and Medicine 40(3): 375–383: PubMed PMID: 33620018. Epub 2021/02/24. eng. 10.1080/15368378.2021.1891092. 33620018

[18] Wang, H. , Y. Xiao , L. Wu , and D. Ma . 2018. “Comprehensive Circular RNA Profiling Reveals the Regulatory Role of the circRNA‐000911/miR‐449a Pathway in Breast Carcinogenesis.” International Journal of Oncology 52(3): 743–754: PubMed PMID: 29431182. Pubmed Central PMCID: PMC5807038. Epub 2018/02/13. eng. 10.3892/ijo.2018.4265. 29431182 PMC5807038

[19] Chen, L.‐Y. , Z. Zhi , L. Wang , Y.‐Y. Zhao , M. Deng , Y.‐H. Liu , Y. Qin , et al. 2019. “NSD2 Circular RNA Promotes Metastasis of Colorectal Cancer by Targeting miR‐199b‐5p‐mediated DDR1 and JAG1 Signalling.” The Journal of Pathology 248(1): 103–115: PubMed PMID: 30666650. Epub 2019/01/23. eng. 10.1002/path.5238. 30666650

[20] Li, D. , M. Masiero , A. H. Banham , and A. L. Harris . 2014. “The Notch Ligand JAGGED1 as a Target for Anti‐tumor Therapy.” Frontiers in Oncology 4: 254: PubMed PMID: 25309874. Pubmed Central PMCID: PMC4174884. Epub 2014/10/14. eng. 10.3389/fonc.2014.00254. 25309874 PMC4174884

[21] Yao, Y. , X. Li , L. Cheng , X. Wu , and B. Wu . 2021. “Circular RNA FAT Atypical Cadherin 1 (circFAT1)/microRNA‐525‐5p/spindle and kinetochore‐associated Complex Subunit 1 (SKA1) Axis Regulates Oxaliplatin Resistance in Breast Cancer by Activating the Notch and Wnt Signaling Pathway.” Bioengineered 12(1): 4032–4043: PubMed PMID: 34288822. Pubmed Central PMCID: PMC8806415. eng. 10.1080/21655979.2021.1951929. 34288822 PMC8806415

[22] Chen, Y. , Z. Li , M. Zhang , B. Wang , J. Ye , Y. Zhang , D. Tang , et al. 2019. “Circ‐ASH2L Promotes Tumor Progression by Sponging miR‐34a to Regulate Notch1 in Pancreatic Ductal Adenocarcinoma.” Journal of Experimental and Clinical Cancer Research 38(1): 466. 10.1186/s13046-019-1436-0. 31718694 PMC6852927

[23] Jia, Y. , and W. Gu . 2021. “Up‐regulation of circPVT1 in T Cell Acute Lymphoblastic Leukemia Promoted Cell Proliferation via miR‐30e/DLL4 Induced Activating NOTCH Signaling.” Pathology, Research & Practice 224: 153536: PubMed PMID: 34237615. Epub 20210623. eng. 10.1016/j.prp.2021.153536. 34237615

[24] Huang, Z. , Y. Ding , L. Zhang , S. He , Z. Jia , C. Gu , T. Wang , et al. 2020. “Upregulated circPDK1 Promotes RCC Cell Migration and Invasion by Regulating the miR‐377‐3P‐NOTCH1 Axis in Renal Cell Carcinoma.” OncoTargets and Therapy 13: 11237–11252: PubMed PMID: 33173313. Pubmed Central PMCID: PMC7648593. Epub 20201103. eng. 10.2147/ott.s280434. 33173313 PMC7648593

[25] Feng, H. , F. Li , and P. Tang . 2021. “Circ_0000745 Regulates NOTCH1‐mediated Cell Proliferation and Apoptosis in Pediatric T‐cell Acute Lymphoblastic Leukemia Through Adsorbing miR‐193b‐3p.” Hematology 26(1): 885–895: PubMed PMID: 34753401. eng. 10.1080/16078454.2021.1997197. 34753401

[26] Gong, G. , J. She , D. Fu , D. Zhen , and B. Zhang . 2022. “CircUBR5 Acts as a Cerna for miR‐1179 to up‐regulate UBR5 and to Promote Malignancy of triple‐negative Breast Cancer.” American Journal of Cancer Research 12(6): 2539–2557: PubMed PMID: 35812044. Pubmed Central PMCID: PMC9251684. Epub 20220615. eng. 35812044 PMC9251684

[27] Li, W. J. , X. X. Xie , J. Bai , C. Wang , L. Zhao , and D. Q. Jiang . 2018. “Increased Expression of miR‐1179 Inhibits Breast Cancer Cell Metastasis by Modulating Notch Signaling Pathway and Correlates with Favorable Prognosis.” European Review for Medical and Pharmacological Sciences 22(23): 8374–8382: PubMed PMID: 30556878. Epub 2018/12/18. eng. 10.26355/eurrev_201812_16535. 30556878

[28] Chen, L. , X. Yang , J. Zhao , M. Xiong , R. Almaraihah , Z. Chen , and T. Hou . 2020. “Circ_0008532 Promotes Bladder Cancer Progression by Regulation of the miR‐155‐5p/miR‐330‐5p/MTGR1 Axis.” Journal of Experimental and Clinical Cancer Research 39(1): 94. 10.1186/s13046-020-01592-0. 32460831 PMC7251916

[29] Gao, P. , X. Zhao , K. Yu , and Z. Zhu . 2021. “Circ_0084582 Facilitates Cell Growth, Migration, Invasion, and Angiopoiesis in Osteosarcoma via Mediating the miR‐485‐3p/JAG1 Axis.” Frontiers in Genetics 12: 690956: PubMed PMID: 34421997. Pubmed Central PMCID: PMC8375504. Epub 2021/08/24. eng. 10.3389/fgene.2021.690956. 34421997 PMC8375504

[30] Fu, L. , Q. Chen , T. Yao , T. Li , S. Ying , Y. Hu , and J. Guo . 2017. “Hsa_circ_0005986 Inhibits Carcinogenesis by Acting as a miR‐129‐5p Sponge and Is Used as a Novel Biomarker for Hepatocellular Carcinoma.” Oncotarget 8(27): 43878–43888: PubMed PMID: 28410211. Pubmed Central PMCID: PMC5546447. eng. 10.18632/oncotarget.16709. 28410211 PMC5546447

[31] Buratin, A. , C. Borin , C. Tretti Parenzan , A. Dal Molin , S. Orsi , A. Binatti , K. Simon , et al. 2023. “CircFBXW7 in Patients with T‐cell ALL: Depletion Sustains MYC and NOTCH Activation and Leukemia Cell Viability.” Experimental Hematology & Oncology 12(1): 12: PubMed PMID: 36681829. Pubmed Central PMCID: PMC9863195. Epub 20230121. eng. 10.1186/s40164-023-00374-6. 36681829 PMC9863195

[32] Shi, Y.‐R. , Z. Wu , K. Xiong , Q.‐J. Liao , X. Ye , P. Yang , and X.‐B. Zu . 2020. “Circular RNA circKIF4A Sponges miR‐375/1231 to Promote Bladder Cancer Progression by Upregulating NOTCH2 Expression.” Frontiers in Pharmacology 11: 605: PubMed PMID: 32457613. Pubmed Central PMCID: PMC7225260. Epub 2020/05/28. eng. 10.3389/fphar.2020.00605. 32457613 PMC7225260

[33] Wei, Y. , X. Chen , C. Liang , Y. Ling , X. Yang , X. Ye , H. Zhang , et al. 2020. “A Noncoding Regulatory Rnas Network Driven by Circ‐Cdyl Acts Specifically in the Early Stages Hepatocellular Carcinoma.” Hepatology 71(1): 130–147. 10.1002/hep.30795. 31148183

[34] Zong, Z.‐H. , Y. Liu , S. Chen , and Y. Zhao . 2020. “Circ_PUM1 Promotes the Development of Endometrial Cancer by Targeting the miR‐136/NOTCH3 Pathway.” Journal of Cellular and Molecular Medicine 24(7): 4127–4135: PubMed PMID: 32073729. Pubmed Central PMCID: PMC7171399. Epub 20200219. eng. 10.1111/jcmm.15069. 32073729 PMC7171399

[35] Li, L. , K. Lei , Y. Lyu , B. Tan , R. Liang , D. Wu , K. Wang , W. Wang , H. Lin , and M. Wang . 2022. “hsa_circ_0001741 Promotes Esophageal Squamous Cell Carcinoma Stemness, Invasion and Migration by Sponging miR‐491‐5p to Upregulate NOTCH3 Expression.” American Journal of Cancer Research 12(5): 2012–2031: PubMed PMID: 35693080. Pubmed Central PMCID: PMC9185627. Epub 20220515. eng. 35693080 PMC9185627

[36] Yao, Y. , X. Chen , H. Yang , W. Chen , Y. Qian , Z. Yan , T. Liao , et al. 2019. “Hsa_circ_0058124 Promotes Papillary Thyroid Cancer Tumorigenesis and Invasiveness Through the NOTCH3/GATAD2A Axis.” Journal of Experimental & Clinical Cancer Research 38(1): 318: PubMed PMID: 31324198. Pubmed Central PMCID: PMC6642504. Epub 20190719. eng. 10.1186/s13046-019-1321-x. 31324198 PMC6642504

[37] Tang, Z. , W. Zhang , A. Liu , C. Wei , M. Bai , J. Zhao , and J. Wang . 2024. “Circ_0104873 Promotes Osteoarthritis Progression via miR‐875‐5p/NOTCH3/Notch Signaling Pathway.” International Journal of Biological Macromolecules 281(Pt 1): 136175: PubMed PMID: 39357702. Epub 20240930. eng. 10.1016/j.ijbiomac.2024.136175. 39357702

[38] Men, X. , A. Hu , and T. Xu . 2024. “CircLZIC Regulates ox‐LDL‐induced HUVEC Cell Proliferation and Apoptosis via Micro‐330‐5p/NOTCH2 Axis in Atherosclerosis.” Clinical Hemorheology and Microcirculation 87(1): 115–127: PubMed PMID: 38277288. Pubmed Central PMCID: PMC11191521. eng. 10.3233/ch-232063. 38277288 PMC11191521

[39] Cong, L. , L. Zhao , Y. Shi , Y. Bai , and Z. Guo . 2024. “Circ_0006476 Modulates Macrophage Apoptosis Through the miR‐3074‐5p/DLL4 Axis: Implications for Notch Signalling Pathway Regulation in Cardiovascular Disease.” Aging 16(16): 11857–11876: PubMed PMID: 39167432. Pubmed Central PMCID: PMC11386933. Epub 2024/08/21. eng. 10.18632/aging.206049. 39167432 PMC11386933

[40] Xu, A. , M. Jiang , S. Li , and Q. Fei . 2020. “Down‐Regulation of circ_0061140 Attenuates Ectopic Endometrial Cell Proliferation, Migration and Invasion in Endometriosis via Inactivating Notch2.” Gene 757: 144926: PubMed PMID: 32621951. Epub 20200702. eng. 10.1016/j.gene.2020.144926. 32621951

[41] Zhang, M. , S. Wang , L. Tang , X. Wang , T. Zhang , X. Xia , and X. Fang . 2019. “Downregulated Circular RNA hsa_circ_0067301 Regulates epithelial‐mesenchymal Transition in Endometriosis via the miR‐141/Notch Signaling Pathway.” Biochemical and Biophysical Research Communications 514(1): 71–77: PubMed PMID: 31023528. Epub 20190423. eng. 10.1016/j.bbrc.2019.04.109. 31023528

[42] Ou, Y. , L. Zhu , X. Wei , S. Bai , M. Chen , H. Chen , and J. Zhang . 2020. “Circular RNA circ_0111277 Attenuates Human Trophoblast Cell Invasion and Migration by Regulating miR‐494/HTRA1/Notch‐1 Signal Pathway in Pre‐eclampsia.” Cell Death & Disease 11(6): 479: PubMed PMID: 32587240. Pubmed Central PMCID: PMC7316814. Epub 2020/06/27. eng. 10.1038/s41419-020-2679-6. 32587240 PMC7316814

[43] Zhang, Y. , T. Gao , X. Li , C.‐C. Wen , X.‐T. Yan , C. Peng , and Y. Xiao . 2021. “Circ_0005075 Targeting miR‐151a‐3p Promotes Neuropathic Pain in CCI Rats via Inducing NOTCH2 Expression.” Gene 767: 145079: PubMed PMID: 32860901. Epub 20200827. eng. 10.1016/j.gene.2020.145079. 32860901

[44] Wu, L. , H. Xu , W. Zhang , Z. Chen , W. Li , and W. Ke . 2020. “Circular RNA circCCDC9 Alleviates Ischaemic Stroke ischaemia/reperfusion Injury via the Notch Pathway.” Journal of Cellular and Molecular Medicine 24(24): 14152–14159. 10.1111/jcmm.16025. 33124180 PMC7753987

[45] Liu, T. , Y. Li , S. Chen , L. Wang , X. Liu , Q. Yang , Y. Wang , et al. 2022. “CircDDX17 Enhances Coxsackievirus B3 Replication Through Regulating miR‐1248/NOTCH Receptor 2 Axis.” Frontiers in Microbiology 13: 1012124: PubMed PMID: 36338034. Pubmed Central PMCID: PMC9627658. Epub 2022/11/08. eng. 10.3389/fmicb.2022.1012124. 36338034 PMC9627658

[46] Si, X. , H. Zheng , G. Wei , M. Li , W. Li , H. Wang , H. Guo , et al. 2020. “circRNA Hipk3 Induces Cardiac Regeneration After Myocardial Infarction in Mice by Binding to Notch1 and miR‐133a.” Molecular Therapy Nucleic Acids 21: 636–655: PubMed PMID: 32736292. Pubmed Central PMCID: PMC7393325. Epub 20200627. eng. 10.1016/j.omtn.2020.06.024. 32736292 PMC7393325

[47] Shi, Q. , J. Wang , Z. Yang , and Y. Liu . 2020. “CircAGFG1modulates Autophagy and Apoptosis of Macrophages Infected by Mycobacterium tuberculosis via the Notch Signaling Pathway.” Annals of Translational Medicine 8(10): 645: PubMed PMID: 32566582. Pubmed Central PMCID: PMC7290638. eng. 10.21037/atm.2020-20-3048. 32566582 PMC7290638

[48] Guo, Z. , X. Manlin , Z. Yanfang , L. Qianxin , L. Fubin , S. Jing , et al. 2021. “Circular RNA Hsa_circ_0006766 Targets MicroRNA miR‐4739 to Regulate Osteogenic Differentiation of Human Bone Marrow Mesenchymal Stem Cells.” Bioengineered 12(1): 5679–5687. 34524066 10.1080/21655979.2021.1967712 PMC8806466

[49] Li, N. , D. Zhang , H. Guo , Q. Yang , P. Li , and Y. He . 2022. “Inhibition of circ_0004381 Improves Cognitive Function via miR‐647/PSEN1 Axis in an Alzheimer Disease Mouse Model.” Journal of Neuropathology and Experimental Neurology 82(1): 84–92: PubMed PMID: 36409993. Epub 2022/11/22. eng. 10.1093/jnen/nlac108. 36409993

[50] Sanadgol, N. , J. Amini , C. Beyer , and A. Zendedel . 2023. “Presenilin‐1‐Derived Circular RNAs: Neglected Epigenetic Regulators with Various Functions in Alzheimer's disease.” Biomolecules 13(9): 1401: PubMed PMID: 37759801. Pubmed Central PMCID: PMC10527059. Epub 2023/09/28. eng. 10.3390/biom13091401. 37759801 PMC10527059

[51] Mei, X. , X.‐B. Cui , Y. Li , and S.‐Y. Chen . 2021 Dec. “CircSOD2: a Novel Regulator for Smooth Muscle Proliferation and Neointima Formation.” Arteriosclerosis, Thrombosis, and Vascular Biology 41(12): 2961–2973: PubMed PMID: 34670409. Pubmed Central PMCID: PMC8612990. Epub 2021/10/22. eng. 10.1161/atvbaha.121.316911. 34670409 PMC8612990

[52] Jin, Y. D. , Y. R. Ren , Y. X. Gao , L. Zhang , and Z. Ding . 2019. “Hsa_circ_0005075 Predicts a Poor Prognosis and Acts as an Oncogene in Colorectal Cancer via Activating Wnt/β‐catenin Pathway.” European Review for Medical and Pharmacological Sciences 23(8): 3311–3319: PubMed PMID: 31081084. Epub 2019/05/14. eng. 10.26355/eurrev_201904_17693. 31081084

[53] Yang, X. , H. Song , Z. Zi , J. Kou , S. Chen , Y. Dai , J. Wang , L. Yuan , and K. Gao . 2019. “Circ_0005075 Promotes Hepatocellular Carcinoma Progression by Suppression of microRNA‐335.” Journal of Cellular Physiology 234(12): 21937–21946: PubMed PMID: 31054187. Epub 2019/05/06. eng. 10.1002/jcp.28757. 31054187

[54] Newman, M. , L. Wilson , G. Verdile , A. Lim , I. Khan , S. H. Moussavi Nik , S. Pursglove , G. Chapman , R. N. Martins , and M. Lardelli . 2014. “Differential, Dominant Activation and Inhibition of Notch Signalling and APP Cleavage by Truncations of PSEN1 in Human Disease.” Human Molecular Genetics 23(3): 602–617: PubMed PMID: 24101600. Epub 2013/10/09. eng. 10.1093/hmg/ddt448. 24101600

[55] Hurley, E. M. , P. Mozolewski , R. Dobrowolski , and J. Hsieh . 2023. “Familial Alzheimer’s disease‐associated PSEN1 Mutations Affect Neurodevelopment Through Increased Notch Signaling.” Stem Cell Reports 18(7): 1516–1533. 10.1016/j.stemcr.2023.05.018. 37352850 PMC10362499

[56] Hamdy, N. M. , M. B. Zaki , N. I. Rizk , N. M. Abdelmaksoud , M. A. Abd‐Elmawla , R. A. Ismail , and A. I. Abulsoud . 2024. “Unraveling the ncRNA Landscape that Governs Colorectal Cancer: a Roadmap to Personalized Therapeutics.” Life Sciences 354: 122946: PubMed PMID: 39122108. Epub 2024/08/10. eng. 10.1016/j.lfs.2024.122946. 39122108

[57] Sokolov, D. , N. Sharda , A. Banerjee , K. Denisenko , E. B. Basalious , H. Shukla , J. Waddell , N. M. Hamdy , and A. Banerjee . 2024. “Differential Signaling Pathways in Medulloblastoma: Nano‐Biomedicine Targeting Non‐coding Epigenetics to Improve Current and Future Therapeutics.” Current Pharmaceutical Design 30(1): 31–47: PubMed PMID: 38151840. Epub 2023/12/28. eng. 10.2174/0113816128277350231219062154. 38151840

[58] Lobry, C. , P. Oh , M. R. Mansour , A. T. Look , and I. Aifantis . 2014. “Notch Signaling: Switching an Oncogene to a Tumor Suppressor.” Blood 123(16): 2451–2459: PubMed PMID: 24608975. Pubmed Central PMCID: PMC3990910. Epub 2014/03/13. eng. 10.1182/blood-2013-08-355818. 24608975 PMC3990910

[59] Chiang, Y.‐F. , K.‐C. Huang , H.‐Y. Chen , N. M. Hamdy , T.‐Chin Huang , H.‐Yi Chang , T.‐M. Shieh , Y.‐J. Huang , and S.‐M. Hsia . 2024. “Hinokitiol Inhibits Breast Cancer Cells in Vitro Stemness‐Progression and Self‐Renewal with Apoptosis and Autophagy Modulation via the CD44/Nanog/SOX2/Oct4 Pathway.” International Journal of Molecular Sciences 25(7): 3904: PubMed PMID: 38612715. Pubmed Central PMCID: PMC11011552. Epub 2024/04/13. eng. 10.3390/ijms25073904. 38612715 PMC11011552

[60] Anwar, M. M. , C. Albanese , N. M. Hamdy , and A. S. Sultan . 2022. “Rise of the Natural Red Pigment 'Prodigiosin' as an Immunomodulator in Cancer.” Cancer Cell International 22(1): 419: PubMed PMID: 36577970. Pubmed Central PMCID: PMC9798661. Epub 2022/12/29. eng. 10.1186/s12935-022-02815-4. 36577970 PMC9798661

[61] Atta, H. , N. Alzahaby , N. M. Hamdy , S. H. Emam , A. Sonousi , and L. Ziko . 2023. “New Trends in Synthetic Drugs and Natural Products Targeting 20S Proteasomes in Cancers.” Bioorganic Chemistry 133: 106427: PubMed PMID: 36841046. Epub 2023/02/26. eng. 10.1016/j.bioorg.2023.106427. 36841046

[62] Golde, T. E. , E. H. Koo , K. M. Felsenstein , B. A. Osborne , and L. Miele . 2013. “γ‐Secretase Inhibitors and Modulators.” Biochimica et Biophysica Acta 1828(12): 2898–2907: PubMed PMID: 23791707. Pubmed Central PMCID: PMC3857966. Epub 2013/06/26. eng. 10.1016/j.bbamem.2013.06.005. 23791707 PMC3857966

